# Analyzing dynamic coupling and coordination of modern service and advanced manufacturing industries in Guangdong–Hong Kong–Macao Greater Bay Area

**DOI:** 10.1016/j.heliyon.2023.e16565

**Published:** 2023-05-23

**Authors:** Chunchun Hu

**Affiliations:** Business School, Shaoguan University, Shaoguan, Guangdong 512005, China

**Keywords:** Guangdong–Hong Kong–Macao Greater Bay Area, Coupling coordination degree, Stock and increment, Modern service industry, Advanced manufacturing industry

## Abstract

Integrating the modern service industry with the advanced manufacturing industry is an important way to cultivate a modern industrial system and achieve high-quality development of economy. This study aims to enhance the supporting and leading role of the Greater Bay Area of Guangdong, Hong Kong, and Macao in China's national economic development and opening up by promoting the in-depth integration of these two industries. A new monitoring system is developed to simulate the level of integration of the modern service and advanced manufacturing industries in the Guangdong–Hong Kong–Macao Greater Bay Area. The dynamic comprehensive evaluation model of stock increment was used to simulate the coupling coordination degree of these two industries. Empirical results reveal that the development of these industries has uneven stock and incremental resource advantages, and their development has not been balanced. The coupling coordination degree of these industries in some areas has become maladjusted, while in other areas, it has fluctuated or developed from coordination to disorderly leap-forward. These findings demonstrate that the study methods and results are significant for analyzing industrial integration processes and promoting in-depth integration development in the Guangdong–Hong Kong–Macao Greater Bay Area. In conclusion, building a monitoring system using the dynamic comprehensive evaluation model of stock increment is an effective way to evaluate the level of integration of these two industries and promote their in-depth integration in the Greater Bay Area.

## 1 Introduction

Integrating the modern service and advanced manufacturing industries is one important way to adapt to the new era of scientific and technological revolution and industrial transformation. It strengthens the core competitiveness of the manufacturing industry, cultivates a modern industrial system, and achieves high-quality development with regard to economy. Prior research has emphasized that choosing an integrated development path is essential for expanding the modern industrial system, extending industry boundaries, and improving industry competitiveness [1]. However, compared to developed countries, there is still a large gap in the level of integration between these two industries [2,3]. In recent years, the trend of integrating the two industries has become increasingly obvious with continuous innovations in new-generation information technology, represented by concepts such as “Internet plus,” big data, blockchain, and others.

The Guangdong–Hong Kong–Macao Greater Bay Area plays an important strategic role in the overall development of the country, with a high degree of cooperation regarding infrastructure, investment and trade, financial services, technology and education, leisure tourism, ecological and environmental protection, and social services. In recent years, the cooperation between its component regions Guangdong, Hong Kong, and Macao has deepened, and the economic strength and regional competitiveness of the Greater Bay Area have improved significantly. However, there are still some problems in the integration of the two industries, such as unbalanced development, weak synergy, insufficient integration depth, and constraints imposed by policy and institutional mechanisms. Hence, there is still a huge potential for developing the integration of the two industries. To accelerate the integrated development of the Greater Bay Area, the Chinese state has introduced relevant policies and measures.

Currently, analyzing the depth of integration between the two industries in the Greater Bay Area has become the focus of much scientific research and national and local strategic decision-making. Developing a scientific monitoring and evaluation index system and measurement model is essential to measure the degree of integration between the two sectors. Since this is a relatively new research field, it is necessary to establish a scientific evaluation system and select appropriate measurement models. This study aims to systematically review existing literature on the evaluation index system used by the modern service and advanced manufacturing industries, sort out the construction of this system, and suggest new ideas for constructing an effective monitoring and evaluation system. Based on these new identified ideas, the study builds a measurement model for measuring, evaluating, and analyzing integrated development between the modern service and advanced manufacturing industries in the Greater Bay Area. The study also provides valuable countermeasures and suggestions to address the current problems in the integration of the two industries.

## 2 Literature review

Industrial convergence refers to the blurring of boundaries between industries, often caused by changes in value proposition, scientific and technological innovation, and market convergence. This phenomenon can lead to industry segmentation [[4], [5], [6], [7], [8], [9], [10]]. Recent research on industrial convergence has emphasized constructing a multi-index comprehensive evaluation system with different factors, including dimensions of scale, potential, innovation and relevance, efficiency, power, environmental trends, and others [[10], [11], [12], [13], [14], [15], [16], [17], [18], [19], [20], [21]]. Previous research has also analyzed the levels of industrial integration across different time and spatial scales by analyzing the development and changing characteristics of industrial integration [[11], [12], [13]]. This was done by analyzing the integrated development levels and coupling coordination degrees of various industries [[14], [15], [16], [17], [18], [19], [20], [21]]. Several methods have been used in this area, including the Herfindahl index method [[22], [23], [24], [25], [26]], the comprehensive index method for assessing industrial integration development [18,27], the coupling coordination degree model method [14,20,[28], [29], [30], [31], [32]], and input-output analysis [[27], [33], [34]].

However, the construction of a multi-index comprehensive evaluation system still faces some issues. For example, when different second-level indicators are set under the same first-level indicators, the comparability of the level of industrial integration development becomes questionable, and similar indicators are repeated. Therefore, it is necessary to achieve incompatibility and weak correlations among relevant indicators. Furthermore, there is a lack of relevant theoretical bases and selection criteria.

Despite the large-scale research on industrial integration, limited research has been conducted on the industrial integration development of the Greater Bay Area. The coupling coordination degree model method is recommended because of the correlations and coordination among the evaluation systems, which reflect the quality of the coordination status. However, the current research has focused on static analysis and rarely uses the “stock increment” index to highlight the dynamic characteristics of index evaluation.

To address this gap, this study constructs a new index system of industrial integration and introduces a coupling coordination model of dynamic comprehensive evaluation level of incremental stock to analyze the development process of industrial integration. The study measures the coupling coordination degree of modern service and advanced manufacturing industries in the Greater Bay Area in 2021, analyzes the integration progress of the two industries, and provides additional reference data for further research on the integration development of the two industries in the Greater Bay Area.

## 3 Methodology

### 3.1 Construction of a monitoring and evaluation index system

#### 3.1.1 Principles for building a monitoring and evaluation index system

To build an effective monitoring and evaluation index system for the two above-discussed industries, we must follow the following principles.

(1) Scientific: The evaluation system should focus on the high-end link of the industrial chain, with emphasis on the innovative applications of new-generation information technology, inter-industry value chain integration, low energy consumption, full value chains, and market business interactions. All relevant indicators should follow scientific and objective principles.
(2) Comparability: The first-level indicators constructed for the development of industrial integration are consistent, and the second-level indicators established for different industries under the first-level indicators should use the same index system to ensure comparability.
(3) Availability: Accurate and objective data is the basis of comprehensive evaluation of industrial integration and development. Therefore, the construction of any index should be based on obtaining real data. Indicators without any data guarantees should not be constructed as far as possible. While constructing an industrial indicator system for the Greater Bay Area, the data channels available in different regions should be considered. The interpretation of such data should be strengthened, and an indicator system with data guarantees should be selected.
(4) Comprehensiveness: The index system should involve all aspects of deep integration and development in the modern service and advanced manufacturing industries, reflecting the process and depth of integration between the two industries. However, indicators with high correlations, similar connotations, or inclusion relationships should be avoided solely for the sake of achieving comprehensiveness.

#### 3.1.2 The construction of a monitoring and evaluation index system

This study constructs a monitoring and evaluation index system to assess the integrated development of the modern service and advanced manufacturing industries in the Greater Bay Area. The index system comprises three categories: the whole value chain indicators, the innovation indicators, and market development indicators (total: 14 detailed indicators, as shown in Table 1).

(1) The whole value chain indicators

**Table 1:** Index system for integrated development of the modern service and advanced manufacturing industries.

Level-1 indicator	Level-2 indicator	Level-3 indicator	Meaning
The whole value chain indicators	Index of production factor input	Labor input	Employment number
		Capital input	Fixed asset investment
		Technical input	R&D investment
	Value creation and value-added index	Labor productivity	Industrial added value/industrial employment number
		Industrial contribution rate	Industrial added value increment/GDP increment
		Industrial pulling force	GDP growth rate × industrial contribution rate
		The effect coefficient of fixed asset investment	Industrial added value increment/industrial fixed asset investment amount
Innovation indicators	Innovation input index	R&D investment	Number of R&D employees
	Innovation output index	Patent applications	Number of patent applications
		Patent ownership rate	Number of patents in force/Number of R&D employees
Market development indicators	Market development index	Number of enterprises	Number of enterprises
		Industrial added value	Industrial added value
		The average remuneration of employed persons	Total wage/employed personnel
		Proportion of industrial added value in GDP	Industrial added value/GDP
		Industrial growth rate	Total output value of the current yea/total output value of the previous year-1

The two selected industries are located at the high end of the industrial chain, and their integrated development is primarily characterized by the significantly enhanced relevance of the value chain in these industries. The advanced manufacturing industry's value-adding activities include research and development, design, production operation, procurement and distribution, and human resource management. Similarly, the modern service industry's value-adding activities comprise e-commerce logistics and distribution services, marketing channel expansion, consulting and after-sales services, and human resource services. These industries' activities are closely intertwined, and the continuous decomposition and integration of the two industrial value chains can make industrial positioning unclear. Therefore, the whole value chain indicators should mainly focus on the input of production factors, as well as value creation and value-added indicators.

The index of production factor input is a relevant factor in this regard. The integrated development of value activities in the modern service and advanced manufacturing industries involves the continuous embedding of the two industries in production factors such as labor, capital, and technology. Therefore, the production factor input index of the whole value chain indicators includes labor, capital, and technology inputs, which are measured using “employment number,” “fixed asset investment” and “R&D investment,” respectively.

Furthermore, the role of value creation and value-added indexes must be discussed. The modern service and advanced manufacturing industries often reconstruct and integrate value chains to realize the integration of the two industries' value chains and create higher customer value. This leads to the growth of enterprise economic performance, improvement of basic production efficiency, enhancement of labor productivity, strengthening of industrial pulling force, increase of investment efficiency, and other value-added activities.

Value creation and value-added indicators include labor productivity, industrial contribution rate, industrial pulling force, and the effect of the coefficient of fixed asset investment; the factors are measured using “industrial added value/industrial employment number,” “industrial added value increment/GDP increment,” “GDP growth rate × industrial contribution rate,” and “coefficient of fixed asset investment effect = industrial added value increment/industrial fixed asset investment amount,” respectively.

(2) Innovation indicators

The modern service and advanced manufacturing industries have emerged in response to the new era of information technology and scientific and technological revolution. Both industries emphasize the innovative application of new-generation information technologies in manufacturing and service industries. Innovation can provide a much-needed competitive advantage, either by improving methods and technologies for creating new products and services or by refining existing ones. Therefore, the innovation index mainly selects innovation input and output as the two indicator categories.

Regarding the role of the innovation input index, R&D investment can be a good indicator of a company's innovation efforts. The innovation input index encompasses the number of R&D personnel and is measured using the number of R&D employees.

Regarding the role of innovation output index, a patent represents the level of innovation and quality of a given innovative product. The index of innovation output encompasses patent applications and patent ownership rates. These factors are measured using “number of patent applications” and “number of effective patents/the number of R&D employees,” respectively.

(3) Market development indicators

As the new age of technological revolution has advanced, the homogenous product markets have become saturated. To seek development, different industries actively develop new products or services, which create newer market demands within their original industries, meeting ever-strengthening consumer demand for personalized experiences. As a result, a more rational market structure has emerged that is conducive to competition, cooperation, and performance improvement, leading to market integration.

Regarding market development indicators, it should be noted that the modern service and advanced manufacturing industries are continuously developing and expanding. The scales of both industries' markets have expanded, industrial functions have been refined, and product and service transactions have been strengthened almost simultaneously. The two industries' businesses exchanges are also increasing, and the relevant market organizations are continuing to converge. With the increasing complexity of industrial chains, the spillover benefits of human, financial, and material resources have begun to emerge, resulting in the emergence of closer industrial relations and a more rational industrial structure. The integrated development of the modern service and advanced manufacturing industries will further enhance professional advantages, resulting in overall benefits for the whole industrial system in the Greater Bay Area. This will lead to a gradual realization of benefit sharing, where both industries will contribute to and benefit from each other's growth.

Market development indicators include input scale, output scale, industrial structure, and the growth rate of output value. These mainly include the number of enterprises, industrial added value, the average remuneration of employed persons, and industrial growth rate, which are measured using “number of enterprises,” “industrial added value,” “total wage/employed personnel,” and “proportion of industrial added value in GDP” ([(total output value of the current year/total output value of the previous year)-1] × 100%), respectively.

### 3.2 Research method

#### 3.2.1 Construction of a comprehensive evaluation model

**Step 1:** Introduction of the Stock Increment Index.

Traditional industry evaluation methods tend to use static evaluation assessments and rarely reflect the changes in the different development stages. To address this, the Stock Increment Index systematically measures the resource base, growth rate, and source of growth impetus of the industry under evaluation [35]. The index evaluates trends in industrial development and highlights the dynamic characteristics of the evaluation process.

Let the original index of the evaluation system be $X_{sj}$ and $X_{mj}$ (*j* = 1,2, …,n). They are the Jth index values of the modern service and advanced manufacturing industries, respectively. The formula used for calculating the incremental index is given in (1).

(1) $$\Delta X_{sj}=X_{sj}-X_{sj-1},\Delta X_{mj}=X_{mj}-X_{mj-1},j=1,2\cdots n$$

where $\Delta X_{sj}$ and $\Delta X_{mj}$ are the increment values of the Jth index of the modern service and advanced manufacturing industries, respectively, and $X_{sj-1}$ and $X_{mj-1}$ are the values of the last period of $X_{sj}$ and $X_{mj}$, respectively.

**Step 2:** Standardization of the Indicators.

As different indices have different dimensions—that is, the units used for quantifying the quantities of the various indices—they cannot be directly compared, so they must be type consistent and dimensionless. The stock index value and incremental index value derived after consistent and dimensionless are shown in Equations (2), (3).

(2) $$\begin{array}{l} x_{sj}=\left\{ \begin{array}{l} \frac{X_{sj}-\min\left(X_{j}\right)}{\max\left(X_{j}\right)-\min\left(X_{j}\right)}X_{sj}\;is\;a\;\text{positive}\;\text{indicator}\\ \frac{\max\left(X_{j}\right)-X_{sj}}{\max\left(X_{j}\right)-\min\left(X_{j}\right)}X_{sj}\;is\;a\;\text{negative}\;\text{indicator} \end{array}\right.\\ x_{mj}=\left\{ \begin{array}{l} \frac{X_{mj}-\min\left(X_{j}\right)}{\max\left(X_{j}\right)-\min\left(X_{j}\right)}X_{mj}\;is\;a\;\text{positive}\;\text{indicator}\\ \frac{\max\left(X_{j}\right)-X_{mj}}{\max\left(X_{j}\right)-\min\left(X_{j}\right)}X_{mj}\;is\;a\;\text{negative}\;\text{indicator} \end{array}\right. \end{array}$$

(3) $$\begin{array}{l} \Delta x_{sj}=\frac{\Delta X_{sj}-\Delta \min\left(X_{j}\right)}{\Delta \max\left(X_{j}\right)-\Delta \min\left(X_{j}\right)}\\ \Delta x_{mj}=\frac{\Delta X_{mj}-\Delta \min\left(X_{j}\right)}{\Delta \max\left(X_{j}\right)-\Delta \min\left(X_{j}\right)} \end{array}$$

where $\chi_{sj}$ and $\chi_{mj}$ are the stock standardization results of the Jth index of the modern service and advanced manufacturing industries, respectively; $\max\left(X_{j}\right)$ and $\min\left(X_{j}\right)$ are the maximum and minimum values of the Jth stock index, respectively. $\Delta \chi_{sj}$ and $\Delta \chi_{mj}$ are the incremental standardization results of the Jth index of the modern service and advanced manufacturing industries, respectively, and $\Delta \max\left(X_{j}\right)$ and $\Delta \min\left(X_{j}\right)$ are the maximum and minimum incremental values of the Jth index of the modern service and advanced manufacturing industries, respectively.

**Step 3:** Build Information Weight.

The mean square deviation method measures the amount of information carried by the stock and increment. After normalization, the weight of information carried by stock and increment can be obtained using the formula shown in (4).

(4) $$\begin{array}{l} \begin{array}{c} \beta_{cj}=\left(\frac{s_{cj}}{s_{cj}+s_{zj}}\right)/\sum_{j=1}^{n}\left(\frac{s_{cj}}{s_{cj}+s_{zj}}\right)\\ \beta_{zj}=\left(\frac{s_{zj}}{s_{cj}+s_{zj}}\right)/\sum_{j=1\left(\frac{s_{zj}}{s_{cj}+s_{zj}}\right)}^{n} \end{array}\\ \begin{array}{c} s_{cj}=\sqrt{\frac{1}{n-1}\sum_{i=1}^{n}{\left(x_{ij}-\frac{1}{n}\sum_{i=1}^{n}x_{ij}\right)}^{2}}\\ s_{zj}=\sqrt{\frac{1}{n-1}\sum_{i=1}^{n}{\left({\Delta x}_{ij}-\frac{1}{n}\sum_{i=1}^{n}x_{ij}\right)}^{2}} \end{array} \end{array}$$

where $\beta_{cj}$ and $\beta_{zj}$ are the weights of the information quantities of stock and increment, respectively, and $s_{cj}$ and $s_{zj}$ are the standard deviations of stock and increment, respectively.

The formula for determining the comprehensive development levels of the two systems’ stock and increment is shown in Equation (5).

(5) $$\begin{array}{l} \beta_{sc}=\sum_{j=1}^{n}\beta_{cj}x_{sj},\beta_{mc}=\sum_{j=1}^{n}\beta_{cj}x_{mj}\\ \beta_{sz}=\sum_{j=1}^{n}\beta_{zj}\Delta x_{sj},\beta_{mz}=\sum_{j=1}^{n}\beta_{zj}\Delta x_{mj} \end{array}$$

where $\beta_{sc}$ and $\beta_{mc}$ are the stock comprehensive development levels of the modern service and advanced manufacturing industries, respectively, and $\beta_{sz}$ and $\beta_{mz}$ are the incremental comprehensive development levels of the modern service and advanced manufacturing industries, respectively.

**Step 4:** Construct a Dynamic Comprehensive Evaluation Level Index.

The formula for calculating the dynamic comprehensive development level index of changes of stock and increment is shown in Equation (6).

(6) $$\varphi_{s}=\alpha \beta_{sc}+\left(1-\alpha \right)\beta_{sz},\varphi_{m}=\alpha \beta_{mc}+\left(1-\alpha \right)\beta_{mz}$$

where $\varphi_{s}$ and $\varphi_{m}$ represent the dynamic comprehensive evaluation level indices of the modern service and advanced manufacturing industries, respectively, *α*∈ [0, 1] is the importance coefficient of stock, (1-*α*) is the importance coefficient of increment, and *α* > 0.5 indicates that the industrial stock factors' contribution to overall industrial development is greater than that of incremental factors. In contrast, *α* < 0.5 indicates that industrial stock factors' contribution to overall industrial development is less than that of incremental factors. In practice, the value can be set according to macro policy or strategic orientation (In this study, we used α as a simulation of a variable and took 0.1, 0.2, 0.3 … 0.8, 0.9, 1, as simulation values.).

#### 3.2.2 Evaluation model for coupling coordination degrees

This study uses the coupling coordination degree model method to measure the coupling coordination degree between the modern service and advanced manufacturing industries’ systems.

(7) $$C=2\times \frac{\sqrt{\varphi_{s}\times \varphi_{m}}}{\left(\varphi_{s}+\varphi_{m}\right)}$$

(8) $$D=\sqrt{C*T}$$

(9) $$T=\beta *\varphi_{s}+\left(1-\beta \right)*\varphi_{m}$$

where C is the degree of coupling of the modern service and advanced manufacturing industries, D is the coupling coordination degree. T is the comprehensive coordination index—a comprehensive evaluation index reflecting the overall synergy or contribution between the two industries, whereas *β* denote undetermined coefficients, which is 0.6 in this study. C, D ranges from 0 to 1; where a higher value indicates higher coherence level between the two systems. The evaluation standard is shown in Table 2.

**Table 2:** Evaluation standard of the coupling coordination degrees between the modern service and advanced manufacturing industries.

D	Coordination Level	$\varphi_{s}>\varphi_{m}$	$\varphi_{s}<\varphi_{m}$
(0–0.1)	Extreme disorder	Advanced manufacturing Industry lag	Modern service Industry lag
(0.1–0.2)	Severe disorder		
(0.2–0.3)	Moderate disorder		
(0.3–0.4)	Mild disorder		
(0.4–0.5)	Near disorder		
(0.5–0.6)	Reluctantly coordinate		
(0.6–0.7)	Primary coordination		
(0.7–0.8)	Intermediate coordination		
(0.8–0.9)	Good coordination		
(0.9–1.0)	Highly coordinated		

## 4 Empirical analysis

### 4.1 Data sources

The data used in this study were mainly sourced from the common statistical yearbook of the nine main cities of the Guangdong Province for the period of 2019–2022, the statistical bulletin for the period of 2018–2021, the statistical website of the Government of the Hong Kong Special Administrative Region, and the website of the Statistics and Census Bureau of the Government of the Macao Special Administrative Region. The Greater Bay Area includes the Hong Kong Special Administrative Region, Macao Special Administrative Region, Guangzhou, Shenzhen, Zhuhai, Foshan, Huizhou, Dongguan, Zhongshan, Jiangmen, and Zhaoqing in the Guangdong Province.

### 4.2 Results and analysis

#### 4.2.1 Dynamic comprehensive evaluation level analysis

As per Equations (1), (2), (3), (4), (5), (6), it is possible to obtain the dynamic comprehensive development levels of the modern service industry (Table 3) and advanced manufacturing industry (Table 4) in the Greater Bay Area for the period of 2021.

**Table 3:** Dynamic comprehensive development levels of the modern service industry in the Guangdong–Hong Kong–Macao Greater Bay Area in 2021.

Area	Stock importance coefficient (*α*)									
	0.1	0.2	0.3	0.4	0.5	0.6	0.7	0.8	0.9	1
Guangzhou	0.344	0.369	0.393	0.418	0.443	0.467	0.492	0.517	0.541	0.566
Shenzhen	0.379	0.413	0.446	0.480	0.513	0.547	0.581	0.614	0.648	0.681
Zhuhai	0.244	0.238	0.231	0.224	0.218	0.211	0.204	0.198	0.191	0.184
Foshan	0.217	0.212	0.207	0.202	0.196	0.191	0.186	0.181	0.176	0.171
Huizhou	0.117	0.116	0.116	0.115	0.115	0.114	0.114	0.114	0.113	0.113
Dongguan	0.224	0.216	0.208	0.200	0.192	0.184	0.176	0.168	0.160	0.152
Zhongshan	0.199	0.198	0.197	0.196	0.195	0.194	0.193	0.192	0.190	0.189
Jiangmen	0.213	0.204	0.196	0.188	0.179	0.171	0.163	0.154	0.146	0.138
Zhaoqing	0.185	0.174	0.163	0.152	0.141	0.130	0.119	0.108	0.096	0.085
Hong Kong	0.250	0.292	0.334	0.376	0.418	0.460	0.502	0.544	0.586	0.628
Macau	0.727	0.679	0.630	0.582	0.533	0.484	0.436	0.387	0.338	0.290

Note: The primary indicator data are obtained from official sources. Then, the dynamic comprehensive development level is calculated by the method of dynamic comprehensive development index (which consists of four steps, see above for details).

**Table 4:** Dynamic comprehensive development level of the advanced manufacturing industry in the Guangdong–Hong Kong–Macao Greater Bay Area in 2021.

Area	Stock importance coefficient (*α*)									
	0.1	0.2	0.3	0.4	0.5	0.6	0.7	0.8	0.9	1
Guangzhou	0.322	0.323	0.324	0.325	0.326	0.327	0.329	0.330	0.331	0.332
Shenzhen	0.352	0.390	0.428	0.466	0.504	0.542	0.580	0.618	0.656	0.694
Zhuhai	0.219	0.221	0.223	0.225	0.227	0.230	0.232	0.234	0.236	0.239
Foshan	0.330	0.337	0.344	0.352	0.359	0.366	0.373	0.381	0.388	0.395
Huizhou	0.501	0.483	0.464	0.446	0.427	0.408	0.390	0.371	0.352	0.334
Dongguan	0.429	0.431	0.432	0.433	0.434	0.435	0.436	0.437	0.438	0.440
Zhongshan	0.241	0.237	0.234	0.230	0.227	0.223	0.220	0.216	0.212	0.209
Jiangmen	0.359	0.342	0.325	0.307	0.290	0.273	0.255	0.238	0.221	0.203
Zhaoqing	0.381	0.356	0.331	0.306	0.280	0.255	0.230	0.205	0.180	0.154
Hong Kong	0.188	0.201	0.213	0.226	0.238	0.251	0.263	0.276	0.288	0.301
Macau	0.504	0.459	0.414	0.369	0.324	0.279	0.233	0.188	0.143	0.098

Note: The primary indicator data are obtained from official sources. Then, the dynamic comprehensive development level is calculated by the method of dynamic comprehensive development index (which consists of four steps, see above for details).

With the increase in the stock importance coefficient from 0.1 to 1, the dynamic comprehensive development level of the modern service industry in Shenzhen, Hong Kong, and Guangzhou showed an upward trend. The dynamic comprehensive development level of the modern service industry in Macao, Zhongshan, Zhuhai, Foshan, Dongguan, Jiangmen, Huizhou, and Zhaoqing showed a downward trend. This industry in Macao showed a large decline, while the industries in the other cities showed a relatively stable decline. The comprehensive development level values are shown in Table 3. This shows that the stock contribution coefficient of the modern service industry in Shenzhen, Hong Kong, and Guangzhou was greater than the incremental contribution coefficient. The three regions accumulated significant advantages in the service industry. They also possessed well-established industrial systems, making them highly competitive in terms of “stock resource” advantages in the modern service industry. The incremental contribution coefficient of modern service industry development in Macao, Zhongshan, Zhuhai, Foshan, Dongguan, Jiangmen, Huizhou, and Zhaoqing was greater than the stock contribution coefficient. The development of the modern service industry had a “late advantage,” which was influenced by policy support, technological innovation, and other factors; furthermore, it also possessed an “incremental resource” competitive advantage.

The dynamic comprehensive development level of the advanced manufacturing industry in Shenzhen, Guangzhou, Zhuhai, Foshan, Dongguan, and Hong Kong showed an upward trend with the increase in the stock importance coefficient from 0.1 to 1. Shenzhen showed a large increase, while the other regions showed stable growth. The comprehensive development level values are presented in Table 4. This shows that the stock contribution coefficient of the advanced manufacturing industry in Shenzhen, Guangzhou, Zhuhai, Foshan, Dongguan, and Hong Kong was greater than the incremental contribution coefficient, indicating a competitive advantage in terms of “stock resources.” The dynamic comprehensive development level of Huizhou, Zhongshan, Jiangmen, Zhaoqing, and Macao was found to be declining, showing that the incremental contribution coefficient of the advanced manufacturing industry in these five regions was greater than the stock contribution coefficient. The late development advantage of the advanced manufacturing industry in these regions was obvious, and the “incremental resource” competitive advantage of this industry was established.

### 4.3 Comprehensive analysis of coupling coordination degrees

#### 4.3.1 Analysis of coupling coordination development levels

The coupling coordination degree between the modern service and advanced manufacturing industries is calculated using Equations (7), (8), (9), and the results are presented in Table 5. The coupling coordination degree reflects the level of coordination between the industries, where a higher value indicates a higher level of coordination.

(1) The coupling coordination degree between the two industries increases with the increase in the stock importance coefficient—the growth trend

**Table 5:** Coupling coordination degree D of the modern service and advanced manufacturing industries in the Guangdong–Hong Kong–Macao Greater Bay Area in 2021.

Stock importance coefficient	0.1	0.2	0.3	0.4	0.5	0.6	0.7	0.8	0.9	1
Guangzhou	0.579	0.591	0.603	0.615	0.626	0.636	0.647	0.656	0.666	0.675
Shenzhen	0.607	0.635	0.662	0.689	0.714	0.738	0.762	0.785	0.807	0.828
Zhuhai	0.483	0.480	0.477	0.474	0.471	0.467	0.464	0.460	0.456	0.452
Foshan	0.507	0.505	0.504	0.502	0.500	0.498	0.496	0.494	0.491	0.489
Huizhou	0.460	0.456	0.452	0.447	0.443	0.438	0.433	0.428	0.423	0.418
Dongguan	0.539	0.533	0.528	0.522	0.516	0.510	0.503	0.497	0.490	0.483
Zhongshan	0.464	0.461	0.459	0.457	0.455	0.453	0.451	0.448	0.446	0.444
Jiangmen	0.512	0.501	0.490	0.478	0.466	0.454	0.441	0.428	0.415	0.401
Zhaoqing	0.497	0.481	0.465	0.448	0.431	0.412	0.393	0.373	0.352	0.329
Hong Kong	0.472	0.501	0.528	0.553	0.577	0.600	0.621	0.642	0.663	0.682
Macau	0.792	0.761	0.729	0.696	0.660	0.622	0.582	0.537	0.488	0.430

Note: According to the data in Table 3, Table 4, the coupling coordination degree was calculated by using the coupling coordination degree evaluation model method.

As the stock importance coefficient increases from 0.1 to 1, the coupling coordination degree between both industries in the Greater Bay Area shows a growth trend. Specifically, the coupling coordination degree of Guangzhou increases from 0.579 to 0.675, indicating a transition from reluctantly coordinating to having a primary coupling coordination. The coupling coordination degree of Shenzhen also increases from 0.607 to 0.828, and the two industries transition from primary coordination to intermediate coordination and then to good coordination. Similarly, the coupling coordination degree of Hong Kong increases from 0.472 to 0.682, and the two industries change from near disorder and reluctant coordination to primary coupling coordination. According to the comprehensive development level of Table 3, Table 4 and in 2021, the development of the modern service industry in Guangzhou, Shenzhen, and Hong Kong is significantly better than that of the advanced manufacturing industry, which lags behind in development.

(2) The coupling coordination degree between the two industries decreases with the increase in the stock importance coefficient—the downward trend

As the stock importance coefficient increases from 0.1 to 1, the coupling coordination degree between industries in the Greater Bay Area decreases, indicating a downward trend. The coupling coordination degree of Zhuhai, Huizhou, and Zhongshan ranges between 0.4 and 0.5, placing the two industries in a state of near disorder. Meanwhile, the coupling coordination degree of Zhaoqing falls between 0.3 and 0.5, classified as mild disorder to near disorder. The development of the advanced manufacturing industry in Zhuhai, Huizhou, Zhongshan, and Zhaoqing is significantly better than that of the modern service industry in 2021, according to the comprehensive development level of Table 3, Table 4.

Foshan, Dongguan, and Jiangmen have a coupling coordination degree between 0.4 and 0.6, indicating that the two industries are changing from reluctant coordination to near disorder. In these three cities, the development of the advanced manufacturing industry has been clearly superior to that of the modern service industry as the stock importance coefficient increases from 0.1 to 1.

However, the coupling coordination degree of Macao decreases from 0.792 to 0.430 with the increasing contribution of stock importance. As a result, the two industries change from intermediate coordination to primary coordination and then to reluctant coordination, eventually transitioning to near disorder. Interestingly, the development of the modern service industry in Macao is obviously superior to that of the advanced manufacturing industry.

#### 4.3.2 Analysis of the coupling coordination mode

(1) Maladjustment mode

The coupling coordination degree between the modern service and advanced manufacturing industries in Zhuhai, Huizhou, Zhongshan, and Zhaoqing is decreasing with the increase in the stock importance coefficient. Both industries have a low development level in the Greater Bay Area, and their role in promoting each other is weak. The two industries are in a state of near disorder and mild disorder, indicating a lack of good coupling interaction between them. This scenario is classified under the maladjustment mode.

However, Zhuhai has taken advantage of the development opportunities of the Greater Bay Area and the Hong Kong Zhuhai Macao Bridge to select the biomedical industry cluster as part of the national strategic emerging industry cluster development project, leading to scientific and technological innovation momentum. Huizhou's advanced manufacturing industries, including electronic communications, computers, medical instruments, and pharmaceutical manufacturing, have maintained a growth trend in recent years. Zhongshan's computer and office equipment manufacturing industry, advanced equipment manufacturing industry, petrochemical industry, biomedicine and high-performance medical equipment industry, new material manufacturing industry, and other advanced manufacturing industries have grown rapidly. Zhaoqing's advanced manufacturing industries, such as new energy vehicles and auto parts, electronic information, biomedicine, and fine chemicals, have developed steadily.

While developing their advanced manufacturing industries, Zhuhai, Huizhou, and Zhongshan have also strived to promote the modern service industry at a new level. However, the development speed of the modern service industry in these cities has been unable to keep up with the development speed of the advanced manufacturing industries. Furthermore, Zhaoqing's industry is still situated in the middle to low end of the value chain, and the development of the modern service industry is lagging behind. The industrial integration and interaction are not close, and the development of the two industries in the four cities has entered a maladjustment mode.

(2) Coordination disorder spanning mode

As the stock importance coefficient increases, Macao's coupling coordination degree, which belongs to the coordination disorder spanning mode, changes from intermediate coordination to primary coordination, then to the reluctant coordinated mode and ultimately to the near disorder mode. Foshan, Dongguan, and Jiangmen have also changed from the reluctant coordinated mode to the near disorder mode.

Macao's economy heavily relies on gambling and tourism, which contributes to more than half of its added value. Although the development of gambling tourism in Macao has grown rapidly in 2021 after a difficult year in 2020, the high dependence of Macao's economy on these services makes it extremely vulnerable. In response, Macao needs to urgently adjust its industrial development strategy and focus on a more moderate and diversified path for economic development.

In contrast, Foshan has been working towards building a global national manufacturing innovation center with 5540 national high-tech enterprises. Dongguan has also accelerated its efforts to build an advanced manufacturing center in the Greater Bay Area, forming a modern industrial system with a complete industrial chain, and electronic information manufacturing as its pillar. Dongguan is home to leading smartphone enterprises like Huawei, OPPO, and Vivo, making it one of the most significant smartphone production bases globally. Jiangmen has focused on building a “six hundred billion industry cluster,” prioritizing high-end equipment manufacturing, new-generation information technology, new materials, new energy vehicles and parts, health, culture, and tourism industries. These cities have seen faster development of advanced manufacturing industries compared to modern service industries, and they have late advantages such as policy support. Incremental resources have become the primary source of power for developing the modern service and advanced manufacturing industries in these regions.

(3) Fluctuation coupling coordination mode

Shenzhen, Guangzhou, and Hong Kong are currently in a fluctuation coupling coordination mode and are undergoing coordinated development with the increase in stock contribution. Shenzhen's coupling coordination degree has changed from primary coordination to intermediate coordination, and then to good coordination. Similarly, Guangzhou's coupling coordination degree has changed from reluctant coordination to primary coupling coordination, and Hong Kong's coupling coordination degree has changed from being near disorder to reluctant coordination, and then to primary coupling coordination.

Shenzhen had an average annual growth rate of GDP exceeding 20% from 1979 to 2021, creating a miracle of urban development on the global stage [36]. Shenzhen remains a leader in innovation and development, and in 2021, its R&D expenditure accounted for 5.49% of its GDP [37]. Shenzhen has ranked among the top of patent units in China in both the number of patent applications and the number of patent grants. The internal structure of the manufacturing and service industries has continued to move towards the medium-high end levels.

In recent years, with the deepening of supply-side structural reform and the emergence of new industries, new business forms, and new models, the service industry has become the driving force and new engine of Guangzhou's economic development. With the emergence of new Internet business forms and models such as sharing economy, platform economy, and network broadcast, the development of information dissemination, software, and information technology service industries has reached new heights. The coupling coordination degree of the modern service industry and advanced manufacturing industry in Guangzhou fluctuates but remains in the coupling coordination state, and the modern service industry has maintained the leading position.

Hong Kong's service industry is an essential pillar of its economy. Hong Kong is a global center of trade, finance, transportation, and commerce, and its service industry has developed rapidly under the unique advantages and strong competitiveness of China's “one country, two systems” policy, and the modern service industry has always maintained a dominant position.

## 5 Discussion and conclusions

This study establishes an evaluation index system for the integrated development of modern service industry and advanced manufacturing industry. Then, it uses the coupling coordination degree model of dynamic comprehensive evaluation level of stock increment to measure the coupling coordination degree of the two industries in the Guangdong–Hong Kong–Macao Greater Bay Area in 2021. The current research results’ implications are as follows.

(1) Differences exist in the advantages of the stock incremental resources used for developing the modern service industry. Shenzhen, Guangzhou, and Hong Kong have benefited from the advantages of stock resource contribution. Other regions have maintained rapid development because of late drivers such as policy and capital innovation. This is a typical advantage of incremental resource contribution. The development of the advanced manufacturing industry also differs across different regions. Shenzhen, Guangzhou, Zhuhai, Foshan, Dongguan, and Hong Kong have formed a relatively complete advanced manufacturing industry system, and they possess the advantages of advanced manufacturing stock resources. Huizhou, Zhongshan, Jiangmen, Zhaoqing, and Macao have promoted the rapid development of the advanced manufacturing industry by relying on policies and other late developmental advantages, and they possess the advantages of advanced manufacturing incremental resources.
(2) As the importance coefficient of stock changes, the coupling coordination degree between the modern service and advanced manufacturing industries in Zhuhai, Huizhou, Zhongshan, and Zhaoqing has become unbalanced. In contrast, Macao, Foshan, Dongguan, and Jiangmen's coupling coordination degree has transformed from coordination to disorderly leap-forward. On the other hand, the coupling coordination degree of Shenzhen, Hong Kong, and Guangzhou has been fluctuating.
(3) The development of modern service and advanced manufacturing industries in the Greater Bay Area is unbalanced. While modern service industry in Guangzhou, Shenzhen, Hong Kong, and Macao is more advanced than advanced manufacturing industry, advanced manufacturing industry in Foshan, Dongguan, Jiangmen, Huizhou, Zhongshan, Zhuhai, and Zhaoqing is more advanced than modern service industry. The coupling coordination degree between the two industries in the area changes with the stock importance coefficient and is not always in the same coordination mode. This finding is important because it differs from previous studies on the integration development of modern service and advanced manufacturing industries in China. Guangdong shows a higher level of integration between the two industries, and the region has been undergoing a good integration stage in recent years. From an industrial development perspective, the development levels of the two industries in the Greater Bay Area are relatively high, but their individual development levels in each region are not equal. The development of one industry is often superior to the other in different regions. Some regions have established modern industrial systems with large bases of modern industrial aggregates and competitive advantages in “stock resources,” while other regions mainly rely on policy support, technological innovation, and other factors to influence their industries and gain a competitive advantage in “incremental resources,” which is a previously unseen development from both static and dynamic perspectives.

### 5.1 Theoretical contributions

This study makes two key contributions to the existing literature. Firstly, it presents a new monitoring and evaluation index system for the integrated development of modern service and advanced manufacturing industries based on three aspects: the whole value chain index, innovation index, and market development index. The construction of this system is informed by relevant theories and standards. Secondly, unlike traditional industrial evaluation methods that focus on static evaluation, this study employs a dynamic comprehensive evaluation index of stock increment to measure the level of integrated development of the modern service and advanced manufacturing industries in the Greater Bay Area. The study evaluates the coupling coordination degree of the two industries from a dynamic perspective.

### 5.2 Limitations and future studies

Although this study has contributed to the research on the coupling coordination degrees of the modern service and advanced manufacturing industries, it has some limitations. Due to data availability limitations, the study could only perform a horizontal analysis of the industrial integration development of 11 cities in the Greater Bay Area for the year 2021, which is insufficient to comprehensively consider the development process. In future studies, a continuous measure of the coupling coordination degrees of the two industries should be established, and the coupling coordination process must be analyzed.

## Funding

This research was funded by the Guangdong Philosophy and Social Science Planning Project “Research on the Monitoring and Evaluation System of the Integrated Development of Modern Service Industry and the Advanced Manufacturing Industry in the Guangdong–Hong Kong–Macao Greater Bay Area” (No: GD20CYJ30).

## Data statements

The data used in this study were collected from the official statistical Yearbook Series and bulletins of Guangzhou, Shenzhen, Zhuhai, Foshan, Huizhou, Dongguan, Zhongshan, Jiangmen, and Zhaoqing in the Guangdong Province, the statistical website of the Government of the Hong Kong Special Administrative Region, and the website of the Statistics and Census Bureau of the Government of the Macao Special Administrative Region.

## Author contribution statement

Chunchun Hu: Conceived and designed the experiments; Performed the experiments; Analyzed and interpreted the data; Contributed reagents, materials, analysis tools or data; Wrote the paper.

## Data availability statement

Data included in article/supplementary material/referenced in article.

## Additional information

No additional information is available for this paper.

## Declaration of competing interest

The authors declare that they have no known competing financial interests or personal relationships that could have appeared to influence the work reported in this paper.
